# Aligning metrics with meaning: considerations for measurement selection in disability evaluation

**DOI:** 10.3389/fresc.2025.1657105

**Published:** 2025-08-14

**Authors:** Elizabeth Marfeo, Elizabeth K. Rasch, Kathleen Coale, Julia Porcino, Leighton Chan

**Affiliations:** ^1^ Department of Community Health, Tufts University, Medford, MA, United States; ^2^ Rehabilitation Medicine Department, NIH Clinical Center, Bethesda, MD, United States

**Keywords:** disability evaluation (MeSH), patient report outcome measure (PRO), work disability, physical function ability, mental health functioning

## Abstract

This article explores the role of patient-reported outcome measures (PROMs) in disability evaluation, a measurement domain traditionally dominated by clinical performance-based assessments. While performance tests are valued for their perceived objectivity, PROMs have gained prominence in research for their efficiency, patient-centered orientation, and capacity to capture subjective experiences relevant to functional decline related to potentially disabling conditions. The manuscript underscores the importance of aligning measurement tools with the specific purpose of evaluation—whether clinical, policy-driven, or programmatic. Using the Work Disability Functional Assessment Battery (WD-FAB) as an illustrative case, it compares the strengths and limitations of PROMs and performance-based tools in evaluating mental and physical function in the context of disability assessment. PROMs such as the WD-FAB can systematically and efficiently generate scores that represent function across multiple domains of function (e.g., mood and emotion, mobility, cognition) and are particularly well-suited for detecting change over time in large-scale applications. In contrast, performance-based assessments, while useful in certain clinical scenarios, are often resource-intensive and may not accurately reflect real-world functioning. The paper argues that although PROMs should not replace performance measures entirely, they represent a valuable and often preferable alternative or complement in many disability evaluation contexts. Ultimately, the choice of assessment tool should consider the intended use, resource constraints, and the need for comprehensive, patient-centered data.

## Introduction

Historically, disability evaluation, whether for benefit determination, programmatic evaluation, or clinical purposes, has focused on performance-based testing due to the general underlying belief that it offers greater value than other forms of measurement such as patient-reported outcome measures (PROMs) (1, 2). However, for research purposes, there has been an overwhelming uptake of PROs with considerable investment in their development, validation, and interpretation in the last decade (3, 4). A substantial body of research has demonstrated the correspondence between performance tests and self-report as well as the conditions under which this correspondence breaks down (5–8). In addition, the advent of symptom-validity tests has helped to address concerns about the intentional misreporting of function for personal gain by offering a way to flag cases of concern (9). This raises questions about why there has not been a better uptake of PROMs for the purpose of disability evaluation. Thus, the goal of this perspective is to elucidate the issues relevant to the use of PROMs in disability evaluation.

## Measurement considerations

The evaluation of disability varies widely based on the purpose of the assessment (10, 11). The selection of the best measurement tool to address a specific scientific question starts with clearly identifying the purpose of measurement. Yet, both clinicians and researchers often gravitate toward discussion of tool availability and features (including psychometric properties) without adequate consideration of what they are trying to accomplish. For instance, if you want to know the number of people who could potentially benefit from a new walker, you need to measure aspects of function among adults who are potential users of the device. Not only would you need to know the number of people with walking difficulties, but if arm use was required for this device, then you would also need to know about arm function and potentially the ability to grip the device. You may need to know about balance and the cognitive ability to operate features of the device. Thus, you need very specific information about a broad range of functioning in the population of interest. In contrast, if you wish to know the population of people who could potentially benefit from civil rights legislation to reduce barriers for people with disabilities, such as the Americans with Disabilities Act, you would want to capture the broadest group of people possible who could be affected by such legislation, including those with difficulties hearing, seeing, thinking, moving, doing everyday activities, and participating in everyday tasks and social activities.

As part of a collaboration with the Social Security Administration, we were tasked with identifying efficient ways to assess work disability. Since contemporary concepts of disability characterize it as the outcome of the interaction between the capabilities of individuals and the environment in which they are operating, the evaluation of work disability requires the examination of all aspects of functioning of the person (at the person level as opposed to the cellular, organ, or body system level) in relationship to the demands of various jobs (12). Thus, by clearly identifying the specific purpose for disability evaluation, the choice of measure, be it self-reported or performance-based, becomes clearer, which simplifies the task of measure selection.

Once measurement options have been narrowed by clarifying its purpose, then other aspects of measurement selection come into play such as content coverage, administration mode (paper/pencil, electronic, interview, telephone), other aspects of administration (observed performance vs. self-report, use of proxy respondents), administration time, cost of administration, the need for trained administrators, and psychometric properties of the instruments. Depending on the situational constraints, more than one type of measure may be selected to best address measurement needs.

One crucial aspect of measure selection that is often overlooked is whether the measure has been tested in real-world situations and if score interpretation has been developed and supported. What good is it to measure the work-related functional capabilities of individuals if the level of ability needed for each functional domain relative to a given job is not known? Real-world studies that provide a foundation for clinical and population-based score interpretation are crucial to selection and use of measures for disability evaluation. Examination of whether normative values have been established, the method for establishing the normative values, the basis for establishing cut-points or thresholds, and comparability of scores over time and across population, are essential considerations of score interpretability.

In the context of measuring functional abilities for the purposes of disability evaluation, assessment, and treatment planning, comprehensive measurement relies on obtaining both the subjective experience of individuals, typically collected via self-report measures, and objective indicators of functioning, which usually rely on performance-based assessments (12). Each measurement approach offers distinct advantages and limitations, which must be considered when interpreting outcomes in the context of disability assessment (13, 14). Performance based tests are especially valuable in clinical settings where decisions about rehabilitation or functional limitations must be grounded in demonstrable performance, however the degree to which that translates into real world functioning in daily activities such as work is limited based on previous research findings (15). Furthermore, when measuring the same underlying construct, self-reported measures and performance-based assessments scores have been found to be often moderate to highly correlated (16). A primary advantage of self-reported measures is their capacity to capture the subjective experience of disability. These self-reported instruments provide insights into how individuals perceive their limitations and how these limitations affect their quality of life and daily activities (17).

To illustrate these advantages and opportunities for improvement in the context of disability assessment, we will draw upon previous research examining the reliability and validity of the Work Disability Functional Assessment Battery (WD-FAB) (18–22). This body of work provides illustrative examples of the advantages and disadvantages of using a robust self-report-based tool to characterize multiple dimensions of physical and mental health function relevant to disability vs. performance-based assessments of disability among populations with underlying physical and/or mental health conditions. The WD-FAB is a standardized self-report measurement tool using item response theory and computer adaptive testing to characterizes physical and mental functional relevant to work disability along eight key dimensions: Basic Mobility; Upper Body Function; Fine Motor Function; Community Mobility; Mood & Emotions; Resilience & Sociability; Self-Regulation; and Communication & Cognition. The WD-FAB also includes an opt-in scale for individuals who use wheelchairs to capture mobility-related tasks. The WD-FAB draws upon a pool of over 300 items to generate scores for all 8 scales in 15–25 min. WD-FAB scores allow direct comparisons between individuals with self-reported activity limitations with the general population of working age adults and are sensitive to detecting change in function over time. See Table 1 for a description of the WD-FAB measurement domains and descriptions of each scale.

**Table 1:** Work disability functional assessment battery (WD-FAB) overview.

The WD-FAB is a 15–20-minute individualized assessment of functional activity that selects test items from a large pool of items to measure self-reported functional ability. The instrument provides scores in the following scales:		
WD-FAB scale	Description	Sample item(s)
Basic mobility	Basic Mobility includes the ability to assume, maintain, and transfer among various body positions and the ability to move around from one place to another	Are you able to bend to look under a car? Are you able to climb 2 or 3 steps up a step ladder?
Upper body function	Upper Body Function includes reaching, lifting, pulling, pushing, and carrying	Are you able to push open a heavy door? Are you able to unload a full grocery cart into a car?
Fine motor function	Fine Motor Function includes manipulation of objects requiring dexterity	Are you able to remove a gas cap from a car?
Community mobility	Community Mobility is defined as using transportation, including public transportation and driving	Are you able to drive in heavy traffic? I can usually get to the bus or train station on time.
Communication & cognition	Communication & Cognition includes aspects of function such as organizational skills, attention, following instructions, oral and written communication	I have trouble putting my thoughts together. Are you able to understand written instructions?
Resilience & sociability	Resilience & Sociability includes aspects of function such as handling stress, accomplishing goals, and learning from mistakes	Please specify your level of agreement: I ask for help when I need to.
Self-regulation	Self-Regulation includes aspects of function such as controlling temper, respecting others, following rules, and social abilities	Please specify your level of agreement: I have difficulty following the rules. Please specify your level of agreement: I am able to work toward long term goals.
Mood & emotions	Mood & Emotions includes aspects of function such as emotional stability, depressive feelings, and anxiety	Please specify your level of agreement: I feel good about myself. In the past 7 days, many situations made me worry.
Wheelchair	The wheelchair scale is an opt-in scale for individuals who use a wheelchair for mobility-related tasks	Are you able to move around in the bathroom, including getting on and off the toilet, from your wheelchair?

## Advantages and disadvantages of PROMs and *in vivo* performance based assessment: illustrative examples using the Work-Disability Functional Assessment Battery (WD-FAB)

### Mental health functioning

One of the key challenges in measurement related to mental and behavioral health is the extreme variation in condition profiles as well as the fluctuating nature of many limitations that may be related to a given mental or behavioral health condition (23). Clinical providers or family members with deep knowledge of a patient often have greater insight into the patient's overall functional ability as compared to the patient themselves. This brings into question how to best capture true functional ability. Almost all mental and behavioral outcomes are derived from some form of self-reported symptoms, impairments, or functional limitations as reported by the patient to the clinical provider. There are few opportunities to observe functional limitations in simulated clinical settings and even fewer opportunities for measurement in real world settings. The area of health outcome measurement for mental health functioning is unique in this way (23).

To examine strengths and weaknesses of performance-based measures vs. self-report measures among individuals with mental or behavioral health conditions, we have use the Vocational Situational Assessment (VSA) is a 35-item assessment used to guide observations and ratings by trained employment providers to measure work performance among individuals with mental and behavioral health disorders (24). To complete the VSA, sites need to simulate a work environment, and employment-specialist raters must be trained to complete the VSA. For VSA implementation, individuals are observed in a natural work setting for at least 1 h per day for a 3–5-day period. Trained raters then keep notes about their observations and provide final scores and ratings at the conclusion of the observation period.

Our previous work examining work-related disability using the WD-FAB vs. clinician reports found that clinical providers and patients have low to fair levels of overall agreement and systematic differences emerge when you examine various dimensions of mental and behavioral health function (25). This was true for both clinician-rated reports of the WD-FAB vs. the patient reported responses on the WD-FAB as well as the concordance with the performance based measure used. For example, in areas that measured aspects of Mood & Emotions, patients’ scores were generally lower than the matched clinical provider scores (26). This indicates that individuals reported that their functional abilities in the area of Mood & Emotions were more severely impaired than how the clinician rated them for the same construct of measurement. In contrast, for areas such as Self-Regulation, the clinician rating was lower than the patient rating. Follow-up qualitative inquiry with the clinical providers suggested this discrepancy related to potential self-awareness or reporting bias in the patient underreporting with impairment in the particular domains that may have more of a socially negative connotation than other domains of mental and behavioral health.

The discordance between provider and patient reporting indicates both perspectives provide valuable insights into underlying functional limitations. In terms of comparing self-report to observational assessments, we found that performance-based assessments were only moderately correlated to self-reported measures provided by both provider and patients. This discrepancy may reflect the underlying limitations of performance-based measures in terms of ecological validity given that these assessments are typically conducted in controlled environments that may not reflect the individual's day-to-day reality. In an ideal setting, both self-report and performance-based assessments would be used to take advantage of the strengths both modalities provide in characterizing a person's mental and behavioral health functioning. However, because many performance-based measures are resource intensive, the feasibility of implementing both is unlikely. Given that self-reported mental health functioning provides a patient-focused description of experiences and functional limitations, this type of reporting may be preferred in many contexts offering significant advantages of cost, time, and accessibility.

### Physical functioning

Using a similar study structure, we examined the utility of using the self-reported WD-FAB as compared to clinician ratings of function and a performance-based functional capacity evaluation called the Physical Work Performance Evaluation (PWPE) among a sample of individuals experiencing physical limitations due to existing musculoskeletal conditions (27). The PWPE is a performance-based assessment that is used in clinical settings to systematically collect information on a person's ability to perform work-relevant tasks. The PWPE involves a range of performance tests consisting of 36 physical tasks that vary in duration. The PWPE must be completed by a licensed clinician and can take approximately 3.5–5 h to complete including preparation, rest periods, and transition/preparation of materials (28).

In contrast to the mental and behavioral health domains, we found that self-reported measures of physical function were more highly correlated with performance-based measures. This finding is consistent with a larger body of research indicating that self-reported measures of physical function are often moderately to highly correlated with performance-based measures of similar constructs of physical function. When thinking about the complexity of disability and work, the WD-FAB offers significant advantages in measurement scope compared to the PWPE. Although the PWPE represents similar tasks and activities to those included in the WD-FAB, the PWPE has a narrower range of tasks given that the assessment must be implemented in a laboratory/clinical setting. This finding again speaks to the limitation of ecological validity of many performance-based assessments. The extent to which these measures translate into real world representation of disability is limited as compared to the subjective experiences of activity limitations and participation restrictions patients describe in navigating their day-to-day lives. This subjective experience is important when looking at overall participation outcomes in the context of disability and is often best captured using self-reported measurement modalities.

In the context of disability evaluation using multiple sources of data, self-reported functioning should be compared to information from other sources to evaluate consistency between the current self-report and behavioral observations, collateral reports, previous self-reports or other records, type and degree of injury/illness, typical symptom presentation, course, response to treatment, etc. (29). Even without these additional sources of data, there are ways to assess the veracity of self-report without any modifications to the test or with minimal modifications. PROMS developed using IRT/CAT based methods such as the WD-FAB instrument can empirically identify discrepancies between obtained scores and expected scores. Mathematical algorithms can help analyze the internal consistency of responses (i.e., answering similar items, similarly, reporting less impairment on easier tasks, more impairment on harder tasks within the same domain). Other approaches to detecting intentional or unintentional misreporting are classified as symptom validity test (SVYs) approaches. Embedded or stand-alone SVTs are measures to assess whether a respondent is providing an accurate and consistent report of his or her symptoms and functioning. Thus, IRT/CAT-based PROMs, such as the WD-FAB, offer a combination of methodological approaches to examine potential misreporting in the form of measurement error by incorporating unscored items, increasing the randomization of item administration, and incorporating symptom validity measures throughout the test.

## Clinical utility and feasibility of integrating performance based and patient reported outcomes in disability evaluation

Recent evidence has emerged to support the collection of both performance-based and patient-reported outcomes measures (PROMs) to evaluate disability across domains at the clinical level. It has been shown that complementary rather than redundant information about a person's functional abilities is captured by implementing both performance-based and PROMs (30, 31). For example many PROMs have been validated with minimal clinically detectable change scores affording standardized and objective longitudinal tracking in the clinical setting (32, 33). The opportunity for improved clinical outcomes by employing PROMs arises from shared decision-making, furthering specificity of care needs and thereby patient engagement in the current era of value-based care. Incorporating PROMs in parallel with performance measures can serve the current triple aim of healthcare if carefully and conscientiously implemented to assess both the individual and population levels. PROMs have been able to capture information affecting overall function previously undisclosed by examining performance-based measures alone (34, 35). Patient perception of performance includes longitudinal retrospection of activity whereas clinical performance measured in a structured, point-in-time environment is limited in its wider applicability to daily function (36). Integrating the two measurement approaches potentially provides insight into whole person function and which domains of function require treatment for return to activity that is meaningful to the patient and measurable by the clinician.

Data from PROMs and evidence-based performance measures is ideally captured at the outset of care to achieve mutually defined goals and expectations for care. PROMs integrated for clinical care have also demonstrated responsiveness over time to further provide guidance for ongoing treatment management (37, 38). Furthermore, PROMS are generally easier and cheaper to obtain, thus more likely to support longitudinal measurement. Given the current emphasis on patient centered care, collecting both relevant patient centered PROMs and performance-based measures in an efficient and timely manner presents challenges in the clinical setting. PROMs can be captured prior to the clinical visit in the EMR portal, via telephone interview, or at the point of care. Considerable resources, including previously implemented software, electronic literacy, health literacy, labor, and proxy availability from the health care setting, provider, and patient are required for PROM integration. Capturing domain information in computer adaptive testing (CAT) tools such as the WD-FAB can ultimately reduce provider and patient burden while improving more specific and individualized data for patient education, shared decision making, goal setting and treatment planning (38). Analysis and interpretation of both PROMs and performance measures at the point of care requires significant clinician focus in busy clinical environments (39). Limited provider time for interpretation and integration of findings from both the PROMs and performance measures is common but necessary to facilitate patient engagement and potentially facilitates self-management of function. Furthermore, when findings from PROMs and performance-based measures are discordant, further analysis may be necessary to specify an efficient and effective treatment plan (40).

Overall, self-report tools such as the WD-FAB provide a way to efficiently collect systematic and comprehensive information about individuals' experiences of their functioning. Self-report measures such as the WD-FAB are not designed to replace medical evidence but are intended to be used to complement and strengthen clinical performance-based measures of health and disability by providing valuable insights in better understanding disability not only as a biomedical issue but as a lived experience. Furthermore, discrepancies between the two types of assessments could prove to be diagnostically meaningful and indicative that further psychological evaluation for complex co-occurring physical and mental health conditions is required. Ultimately selecting between performance-based measures and self-reported measures of health, function and disability requires careful attention to the patient's individual needs, the clinical or research setting, and the practical resources available. Table 2 summarizes the overall strengths and limitations of each measure to consider when thinking about which to use in practice.

**Table 2:** Summary of strengths and limitations of patient reported outcomes and performance measures.

Type of measure	Advantages	Disadvantages
Patient reported outcomes	Person-Centered Approach • Adds person centeredness. • Provides insight into patient's interpretation of their own limitations. • Opportunity for targeted interventions Flexible & Less Resource-Intensive • Ability to monitor/manage patients at a distance. • Assess multiple domains of function in one tool. • Efficiency/can reduce visits and allow others access. Ecological Validity • Reflects the actual environmental conditions in which respondents operate. • Respondents can consider more than one environment in which they typically function. • Respondents can “average” responses over hours/days/weeks (more generalizable) • Easier to obtain longitudinal data	Potential Measurement Reporting error • May be problematic for respondents who do not have good self-awareness. • Could potentially be altered/biased to suit situation (gaming/intentional misreporting)
Performance measures	Reduces error due to reporting bias. Clinical Utility • It can be highly focused on targeting specific body system or impairments. • Highly structured to maximize reliability and validity to guide diagnostic or clinical intervention	Often less patient-centered • Potential for patient fatigue • Mental/emotional processes are not easily observed. Less Comprehensive • Constrained set of measures to reflect domain of interest. • May not account for co-morbidities. Resource Intensive • Often only trained professionals can administer. • Time and clinical setting constraints Limited generalizability • Not reflective of real-world settings • Represents patient's ability at a specific point in time

## Conclusion

Different measures offer different perspectives on health, function, and disability, each with their own benefits and tradeoffs. Performance-based testing supposedly provides a more objective perspective, but it is limited by the environment in which the testing is conducted, which does not often reflect the complexity or variability of real-world situations. Patient-reported outcome measures (PROMs) can be more comprehensive and more closely reflect respondents lived experiences, although there are sometimes concerns around reporting bias, which can be at least partially addressed through measure validation. In the context of disability evaluation, one key consideration is patient burden. PROMs can take less time to complete and can be completed in patients' own environments. This reduces the time, effort, and resources a patient would be required to put forth to make it to a testing site. Ultimately, it is important to use an approach that provides the most relevant measure to the disability evaluation purpose.
